# Exploring the roles of tryptophan metabolism in MS beyond neuroinflammation and neurodegeneration: A paradigm shift to neuropsychiatric symptoms

**DOI:** 10.1016/j.bbih.2021.100201

**Published:** 2021-01-04

**Authors:** Lorraine S.Y. Tan, Heather M. Francis, Chai K. Lim

**Affiliations:** Faculty of Medicine, Health and Human Sciences, Macquarie University, Australia

**Keywords:** Kynurenine pathway, Neuroinflammation, Depression, Fatigue, Cognitive dysfunction, Multiple sclerosis

## Abstract

The metabolism of tryptophan through the kynurenine pathway (KP) has been increasingly recognised in contributing to disease progression in the autoimmune and inflammatory disease multiple sclerosis (MS). In this review, the roles of inflammation and the KP are recontextualised to better understand the aetiology of the neuropsychiatric symptoms (depression, postpartum depression, suicidality, fatigue and cognitive dysfunction) in MS. These symptoms will be discussed in the context of cytokine-induced sickness behaviours, KP activation and levels of neurotoxicity and neuroprotection in MS. In particular, there will be emphasis on how neuropsychiatric symptoms in MS occur against the shared background of inflammation and KP dysregulation. The discourse of this review aims to promote future research in elucidating KP mechanisms in MS that would inevitably lead to more targeted treatment options for neuropsychiatric symptoms and disease progression.

## Highlights

• Research on tryptophan metabolism and neuroinflammation on neurodegeneration in multiple sclerosis (MS) is mounting.
• This review reframes the roles of neuroinflammation and tryptophan metabolism dysregulation on mental health issues in MS.
• The impact of neuroinflammation and tryptophan metabolism on depression, suicidality, fatigue, and cognitive impairment in MS are discussed.

## 1 Introduction

Multiple sclerosis (MS) is an autoimmune, demyelinating disease of the central nervous system (CNS) affecting 2.3 million people worldwide (Multiple Sclerosis International Foundation, 2013). Neuropsychiatric symptoms are some of the most prevalent yet poorly recognised and managed symptoms in MS. Given the disease is often diagnosed in early adulthood (with the average age of onset being 30 years), these pervasive symptoms need to be adequately managed throughout the decades of disease progression. Symptoms such as depression, postpartum depression (PPD), suicidality, fatigue and cognitive impairment have been associated with reduced quality of life and work loss, both of which have significant impacts on a global socioeconomic level. In Australia, the socioeconomic cost of MS is rising, at $1.75 billion in 2017, with loss of wages from people with MS (pwMS) accounting for over a third of the financial burden (Ahmad et al., 2018). These figures represent a substantial burden on pwMS and their families. As such, it is important to understand the mechanisms behind the core symptoms that impact functional outcomes in pwMS so that future treatment options can improve quality of life and utility.

MS is becoming increasingly recognised as a disease with both inflammatory and neurodegenerative mechanisms (Trapp and Nave, 2008). Both have distinct roles that are pathologically related to disease relapse and progression. In recent years, cumulative research has brought attention to the link between neuroinflammation and neurodegeneration through metabolic activation and dysregulation of the kynurenine pathway (KP) of tryptophan metabolism. Indeed, accumulation of neurotoxic KP metabolites has been implicated in depression, and neurocognitive diseases including Alzheimer’s disease, Parkinson’s disease, amyotrophic lateral sclerosis, human immunodeficiency virus (HIV) associated neurological disorders and Huntington’s disease (Lovelace et al., 2017). The purpose of this review is to discuss the role of inflammation and its well established links to cytokine-induced sickness behaviour and KP dysregulation to explain the experience of depression, postpartum depression, suicide, fatigue and cognitive dysfunction in pwMS. This review will be structured around the interplay between inflammation, cytokine-induced sickness behaviour (Section 2.1) and KP dysregulation in MS (Section 2.2). Specifically, each section will outline how these respective mechanisms contribute to each of the following neuropsychiatric symptoms in MS: depression (Section 3), postpartum depression (Section 4), suicidality (Section 5), fatigue (Section 6), and cognitive dysfunction (Section 7). Overall, this review aims to bridge the associations between immune dysfunction, inflammation and the KP to initiate a paradigm shift in understanding KP related mechanisms in contributing to neuropsychiatric symptoms in MS.

## 2 Inflammation and the KP in MS

### 2.1 Cytokine-induced sickness behaviour in MS

Cytokines are involved in the immune and inflammatory processes that contribute to MS pathology such as demyelination, axonal injury, oligodendrocyte death and neuronal dysfunction. In both experimental and clinical research, cytokine production has been shown to fluctuate in parallel with MS disease activity. The increase of pro-inflammatory cytokines interferon gamma (IFN-γ), tumor necrosis factor alpha (TNF-α) and interleukin 6 (IL-6) prior to and during clinical relapse have been widely demonstrated in the literature in relapsing-remitting MS (RRMS) compared to controls (Imitola et al., 2005). On the other hand, during remission phases of MS, there is an increase in anti-inflammatory cytokines such as IL-4 and IL-10 (Imitola et al., 2005).

Pro-inflammatory cytokines are associated with a class of neurobehavioural changes that occur in the event of infection and inflammation, known as cytokine-induced sickness behaviour (Dantzer and Kelley, 2007). These symptoms occur in individuals in the event of infection and inflammation and can be characterised by, but not limited to, lethargy, anhedonia, malaise and a reduction in eating and drinking (Dantzer and Kelley, 2007). Sickness behaviour was initially perceived as an adaptive mechanism to efficiently conserve energy during infection; however, when considering chronic inflammatory conditions, these behavioural responses are often out of proportion to their causal factors (Dantzer and Kelley, 2007). Prolonged sickness behaviour in itself is an effortful expense on metabolic resources. The symptoms of sickness behaviour can be classified as psychological (depressed mood, anxiety and cognitive dysfunction) and/or sedentary behaviours, which include neurovegetative and somatic symptoms (fatigue, insomnia, appetite loss and pain) (Dantzer and Kelley, 2007). Sickness behaviour has also been linked to depression due to the overlap in symptoms between both conditions. Interestingly, people with depression display immune profiles indicative of innate immune system activation (Maes, 1993). In MS, there is also an increase in pro-inflammatory cytokines during acute relapse, which may be a mechanism underlying the neuropsychiatric symptoms that overlap with sickness behaviour.

### 2.2 Inflammation and KP activation in MS

**Fig. 1:**
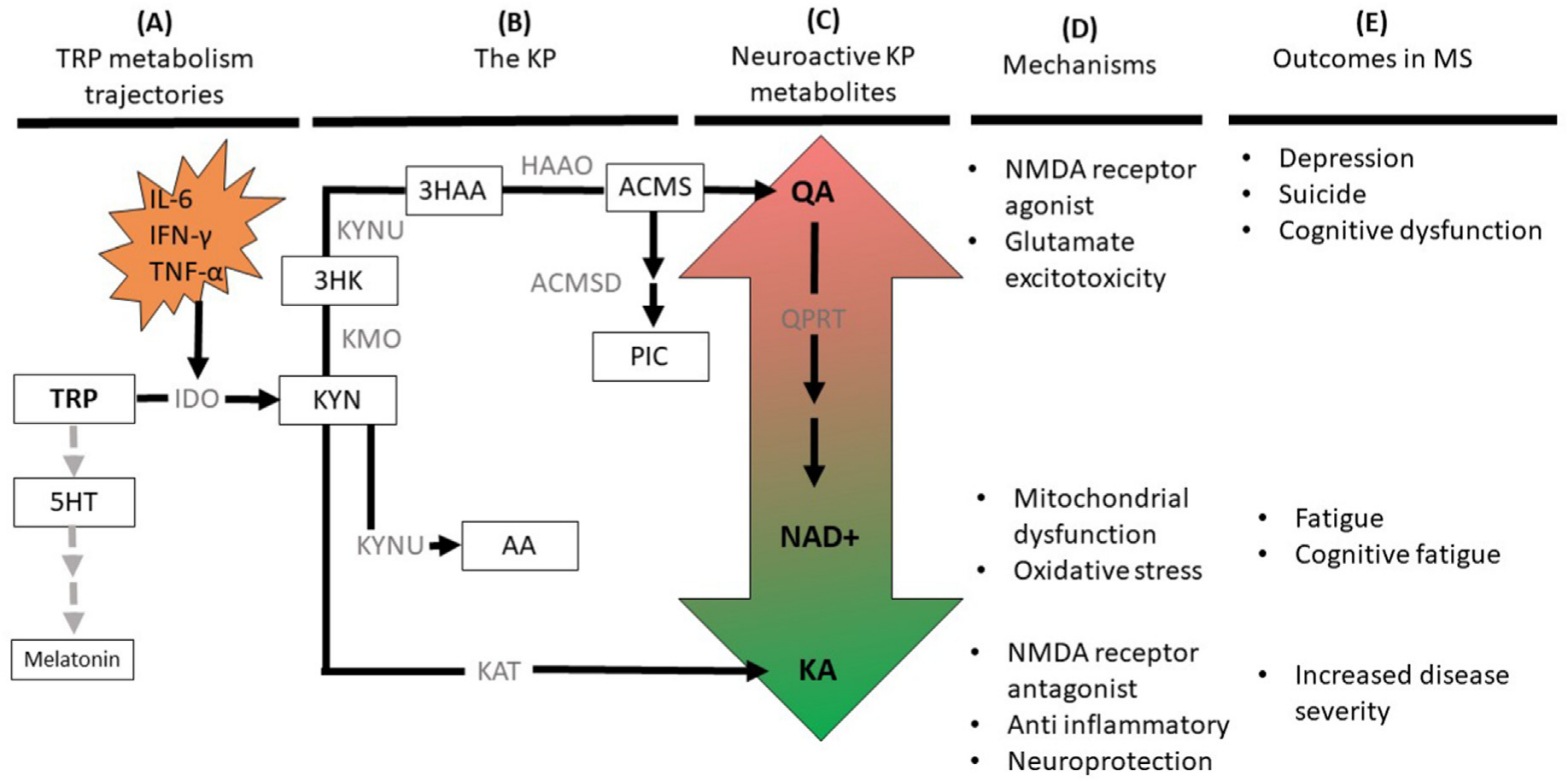
The mechanisms of the KP that determine neuropsychiatric outcomes in MS. This diagram demonstrates the mechanisms of KP metabolism on neuropsychiatric symptoms in MS. From left to right, (A) depicts that the trajectory of TRP metabolism is dictated by pro-inflammatory cytokines to divert 5HT and melatonin production (grey arrows) and promote KP metabolism (B) in MS. The level of neurotoxicity of neuroactive KP metabolites (C) are contrasted by increased levels of neurotoxicity, through higher levels of QA relative to neuroprotective levels of NAD+ and KA levels. As a result, the mechanisms (D) of these imbalances can directly impact NMDA receptors causing glutamate excitotoxicity, impair mitochondrial function, and promote oxidative stress and a pro-inflammatory environment. The corresponding consequences of this contributes to neuropsychiatric symptoms (depression, suicide, fatigue and cognitive dysfunction) in MS and can also impact disease progression (E). IL-6: interleukin-6; IFN-γ: interferon-gamma; TNF-α: tumor necrosis factor-alpha; 5HT: serotonin; TRP: tryptophan; KYN: kynurenine; KA: kynurenic acid; 3HK: 3-hydroxykynurenine; 3HAA: 3-hydroxyanthranilic acid; AA: anthranilic acid; ACMS: 2-amino-3-carboxymuconate-6-semialdehyde; PIC: picolinic acid; QA: quinolinic acid; NAD+: nicotinamide adenine dinucleotide; IDO: indoleamine 2,3-dioxygenase; KAT: kynurenine aminotransferase; KMO: kynurenine monooxygenase; KYNU: kynureninase; HAAO: 3-hydroxyanthranilate 3,4-dioxygenase; ACMSD: 2-amino-3-carboxymuconate-6-semialdehyde decarboxylase; QPRT: quinolinate phosphoribosyltransferase.

The significance of the inflammatory mechanisms in MS is realised through the downstream consequences of KP activation. The KP is responsible for more than 95% of the degradation of the essential amino acid, tryptophan (TRP) to create nicotinamide adenine dinucleotide (NAD+), a crucial component of cellular energy. Cytokines (including IFN-γ and TNF-α) are potent inducers of the first-rate limiting enzyme of the KP, indoalomine-2-3 dioxygenase (IDO) (Kwidzinski et al., 2005). IDO catalyses the conversion of TRP into kynurenine (KYN) and from here, there are three separate pathways of metabolism: conversion into kynurenic acid (KA) through the enzyme kynurenine aminotransferase or quinolinic acid (QA) through either 3-hydroxykynurenine or anthranilic acid pathways (Fig. 1). KA has a neuroprotective role as a glutamate antagonist through binding to N-methyl-D-aspartate (NMDA), α-amino-3-hydroxy-5-methyl-4-isoxazolepropionic acid (AMPA) and α7 nicotinic acetylcholine receptors (Moroni et al., 2012). Conversely, QA is a strong NMDA receptor agonist that can over activate NMDA receptors to increase intracellular Ca2+ levels leading to oxidative stress and cell death through glutamatergic excitotoxicity (Guillemin, 2012). As such, QA promotes excitotoxicity in the CNS by releasing glutamate and glutamine synthase while KA is anti-inflammatory and can facilitate the regulation and inhibition of glutamate release to counteract the neurotoxic effects of QA (Foster et al., 1984).

KP activation via IDO, plays a crucial role in immunoregulation. Increased IDO expression, mediated by pro-inflammatory cytokines, depletes TRP and promotes immune suppression through the proliferation of regulatory T cells (Munn et al., 1999). For example, injection of 1-methyl-tryptophan (an IDO inhibitor) in pregnant mice induces foetal rejection, emphasising the importance of maternal immunosuppression to maintain foetal tolerance during pregnancy (Munn et al., 1998). The same mechanisms of IDO are present during inflammatory states in the context of MS relapse, where immunosuppression is protective in terms of dampening autoimmune responses. IDO activation, as measured through an increased KYN/TRP ratio, is higher in pwRRMS during relapse than in stable phases or during immunosuppressive (glucocorticoid) treatment (Mancuso et al., 2015). Similarly, IDO activation has been detected during preclinical and symptomatic phases of experimental autoimmune encephalomyelitis (EAE), the animal model of MS (Kwidzinski et al., 2005). IDO activity has been shown to be higher in EAE mice during the first remission phase compared to control mice, highlighting its immunoregulatory role in remission in EAE (Sakurai et al., 2002). Moreover, IDO inhibition in mice can exacerbate EAE severity, increase levels of pro-inflammatory cytokines and reduce regulatory T cell responses (Kwidzinski et al., 2005; Yan et al., 2010). As such, disease activity in MS is dictated by IDO levels which are mediated through pro-inflammatory cytokines.

Interestingly, the KP metabolite profile in MS is characterised by a higher QA/KA ratio, which is related to disease progression (Lim et al., 2017). Lower levels of KA are evident in MS while QA concentrations rise progressively with disease severity (Lim et al., 2017; Rejdak et al., 2002). As such, the immunosuppressive potential of initial IDO activation is ultimately counterbalanced by KP dysregulation in MS and implicates TRP metabolism as a mechanism associated with disease severity. In terms of neuropsychiatric symptoms, the role of inflammation and the KP is less established in MS and the following sections will discuss collective evidence from the literature to implicate the role of these mechanisms. We argue that the same pathological mechanisms perpetuating the MS disease course are also behind the hallmark neuropsychiatric symptoms of MS.

## 3 Depression in MS

Depression is highly prevalent, affecting over 40% of individuals in the MS community (Lydia Chwastiak et al., 2002). Much like sickness behaviour, there are psychological (pessimism, guilt, sadness) and sedentary (energy loss and sleep change) components to the symptoms of depression in MS (Sacco et al., 2016). Both are common in MS; psychological symptoms of depression account for a large proportion of symptom variation (35%), however, sedentary symptoms may be linked to fatigue and disability in MS (Sacco et al., 2016). This supports a prevailing argument that neurological and psychiatric symptoms overlap in MS.

### 3.1 Inflammation and depression in MS

The occurrence of depression and its severity appears to be linked to the disease course of MS. Self-reported depression tends to peak during relapses (Koch et al., 2008) and acute attacks, with levels almost three times higher than healthy controls (Kahl et al., 2002). Levels of depression in MS can remain relatively unchanged over 10 years of follow up, which does not resemble an idiopathic pattern (Koch et al., 2008). Further, depression may also worsen disability in MS. A retrospective cohort study found that a diagnosis of depression was the only psychiatric comorbidity that moderated a long-term increase in disability in MS (McKay et al., 2018). This link between depression and disease progression in MS may suggest a shared underlying aetiology.

Supportive of this notion, the lifetime development of depression is higher in pwRRMS than in primary progressive MS cohorts, which may highlight disease course factors such as inflammation in the pathogenesis of depression, through cytokine-induced sickness behaviour. The overlap in inflammatory markers involved in MS and depression supports this argument. Specifically, IL-6 and TNF-α have been identified as the only two cytokines that are consistently elevated in diagnosed major depression (Dowlati et al., 2010). IL-6, TNF-α and IFN-γ in the periphery are moderately related to self-reported depression symptoms during MS relapse, and TNF-α levels during this time are strongly linked to long-term depression at follow up, 3–6 months later (Kahl et al., 2002; Ibrahim and Afifi, 2012). IL-6 levels are also significantly higher in pwMS with depression compared to those without depression (Kallaur et al., 2017). Conversely, clinical trials have established the therapeutic value of non-steroidal anti-inflammatory drugs (NSAID), as an adjunct to antidepressants for individuals with treatment resistant major depression by inhibiting the production of cytokines, namely IL-6 (Faridhosseini et al., 2014). In one trial, adjunct NSAID therapy was beneficial in improving severe depression and able to reduce IDO and IFN-γ levels (Krause et al., 2017). Taken together, these findings suggest an inflammatory basis for mood disturbance that may be targeted to reduce depression in MS.

### 3.2 KP dysregulation and depression in MS

There are both upstream and downstream consequences of KP activation in MS that are associated with depression. Our discussion of the association between KP activation and depression will explore these consequences related to the limited biosynthesis of serotonin (5HT) and melatonin in MS (Fig. 1A) and the impact of KP inducing disease-modifying-drugs on both disease exacerbation and mood to demonstrate how these mechanisms work together to produce the neurological and neuropsychiatric MS symptoms in tandem.

KP activation can limit 5HT production due to the limited bioavailability of TRP as a precursor. Evidence of upstream collateral abnormalities related to TRP metabolism include lower 5HT metabolism in pwMS compared to controls, which is negatively correlated with disability in pwRRMS (Markianos et al., 2009). In fact, there is a negative relationship between 5HT metabolism, disability and rate of axonal loss in RRMS (Markianos et al., 2009). Further, increasing endogenous 5HT levels in EAE mice was efficacious in reducing depressive behaviors and EAE onset (Musgrave et al., 2011). In addition, 5HT is a precursor of the neurotransmitter melatonin, which is important in promoting remyelination in MS, through its anti-inflammatory and anti-oxidant properties. Interestingly, seasonal melatonin deficiency is significantly associated with MS relapse (Farez et al., 2015). Nocturnal serum melatonin levels are further reduced during acute attacks in pwRRMS who are depressed, compared to non-depressed MS controls, a finding consistent with melatonin reductions in major depression (Akpınar et al., 2008). Not only are the depletion of these neurotransmitters well established in depression, but it seems that they are also implicated in MS disease progression. As such, the reductions of 5HT and melatonin, which influence mood and have broader consequences on clinical progression in MS, can be recognised as a secondary consequence of KP activation that can be induced through the shared inflammatory mediators in depression and MS.

The benefits of initial KP activation can minimise inflammation and clinical relapse in EAE (Kwidzinski et al., 2005). This mechanism is utilised by popular immunomodulatory treatments for pwMS, such as interferon-β (IFN-β), to dampen inflammation and enhance KP activation to supress T cell activity. Although, long term use of IFN-β therapy is associated with both increased KYN/TRP ratios (reflecting higher KP activity) (Amirkhani et al., 2005) and stability of depression in MS (Arnett and Randolph, 2006). In fact, higher levels of the KYN/TRP ratio measured in cerebrospinal fluid (CSF) is predictive of clinical depression in newly diagnosed pwMS (Aeinehband et al., 2016). These converging findings highlight the possible treatment limitations of IFN-β in MS due to the counteractive effects of KP activation on depression. Ongoing KP activation can lead to the downstream consequences of QA production (Heyes et al., 1997) and IFN-β administration in human macrophages has demonstrated KP activation increasing QA levels in a dose-dependent manner (Guillemin et al., 2001). It is probable that long-term usage of immunomodulatory therapies may chronically alter KP activity in a manner that exacerbates both disease progression and depression in MS. This highlights the need for a treatment option that can reduce inflammation without aggravating the KP. As discussed, NSAID adjunct treatments have been effective for individuals with major depression, and especially so for those with a higher KYN/TRP profile (Krause et al., 2017). QA production was also reduced in those with resolved depressive symptoms by the end of the trial (Krause et al., 2017), emphasising the relevance of further research on NSAID as an alternative option to mediate inflammatory mechanisms in MS.

### 3.3 Summary of depression in MS

Overall, the prevalence of depression in MS is complex but largely driven by inflammatory mechanisms that affect the duration and severity of symptoms. Depression in MS may be better understood as a cytokine-induced sickness behaviour that is sustained by the bidirectional disruptions to TRP metabolism through 5HT and KP dysregulation. The relationship between inflammatory and immune related factors builds a consistent narrative that depression may be an organic by-product of these processes, which are aberrant in the pathology of MS. What is also concerning is the evidence of current MS treatments exacerbating this process.

## 4 PPD in MS

During pregnancy, immune and inflammatory changes are necessary to protect against foetal rejection and to deliver the baby. As discussed, this corresponds with an increase in IDO activity that is critical in the maintenance of pregnancy. Interestingly, this immunosuppressive mechanism is also apparent in pwMS during pregnancy and may explain why relapse rates are the lowest at this time. Moreover, increases in pro-inflammatory cytokines and KP activation that have been established in depression are also observed during the perinatal period of pregnancy. These changes are thought to cause the phenomenon of PPD, which is the occurrence of depression during the early puerperium, reported in an average of 10–15% (ranging up to 60%) of women (Halbreich and Karkun, 2006). Coincidently, MS relapse rates double within the first three months postpartum, compared to the year before pregnancy (Confavreux et al., 1998). The pro-inflammatory shift that occurs universally postpartum provides mounting evidence that acute mood changes and MS disease exacerbation occur as a result of inflammation and KP activity. The links between these relationships can be used to enhance our understanding of the rates of depression and MS relapse throughout pregnancy.

### 4.1 Inflammation and KP dysregulation in PPD

In early puerperium, pro-inflammatory cytokines (TNF-α and IL-6), are increased in women who self-report anxiety and depression and are predictors of the consistency and long-term risk of mood disturbance (Maes et al., 2002; Boufidou et al., 2009). Similarly, the ratio of pro-inflammatory cytokines (compared to anti-inflammatory cytokines) is significantly higher after birth than during gestation in pregnant women with RRMS (Al-Shammri et al., 2004). While IDO is induced during active disease states in RRMS (Kwidzinski et al., 2005; Mancuso et al., 2015), there is also evidence of rising KP activity in new mothers with PPD, showing a higher KYN/TRP ratio in those with escalated self-reported anxiety and depression in the puerperium (Maes et al., 2002). Suicidal mothers with PPD also have greater pro-inflammatory changes and higher KYN production (relative to 5HT) than healthy controls, indicative of KP activation (Achtyes et al., 2020). The correlation between KYN/TRP ratio and IL-6 in PPD mothers further substantiates that inflammation alters TRP degradation in favour of the KP (Maes et al., 2002). These mechanisms may be more vulnerable in pregnant pwMS, especially in the postpartum period where both acute mood disturbance (PPD) and disease relapse align with IDO and cytokine changes. This provides another account of how depression and disease activity in MS share overlapping mechanisms.

### 4.2 Summary of PPD in MS

Both MS relapse and childbirth are associated with acute pro-inflammatory changes that evidently trigger metabolic abnormalities in TRP degradation to produce measurable declines in mood. Along this line, IDO activity can also dictate the patterns of disease exacerbation and remission during pregnancy in RRMS through balancing cytokine levels (Zhu et al., 2007), however more insight is needed to elucidate the levels of IDO with regards to MS relapse during pregnancy given IDO activity is evident in both relapse and successful pregnancy. Perhaps this may also implicate more severe mood symptoms in new mothers with MS, warranting the need to better understand these concepts within an autoimmune framework.

## 5 Suicidality in MS

Evidence of inflated suicide rates in autoimmune disorders and neuroinflammatory diseases suggest that immune system dysregulation can elicit suicidal behaviour (Brundin et al., 2017). This is especially relevant in MS as the prevalence of suicide is 2–7 times greater than in the general population (Stenager and Stenager, 1992). Intriguingly, completed suicides in MS are not associated with disease burden characteristics such as a higher disability or longer disease duration (Sadovnick et al., 1991). While this is unexpected, pro-inflammatory mechanisms are dominant in RRMS, where rates of depression are highest (Zabad et al., 2005), which strengthens the argument that depressive symptoms are linked to a pro-inflammatory aetiology.

### 5.1 Inflammation and KP dysregulation in suicidality and MS

The contribution of pro-inflammatory cytokines on KP dysregulation have a remarkable impact on suicidal behaviour. Levels of pro-inflammatory cytokines (IL-6 and TNF-α) are higher in suicide attempters compared to non-suicidal depressed controls. This relationship is independent of depression severity and remains even when potential demographic confounders such as age, sex and BMI are controlled (Janelidze et al., 2011). Understanding the inflammatory nature of suicide is pertinent in MS as case studies have identified that during treatment, IFN-β therapy can elicit severe depression with suicidal ideation and attempts in pwRRMS without a previous psychiatric history (Fragoso et al., 2010). While TRP metabolites were not measured in this study, consistent findings of increased KYN/TRP ratios and QA levels following IFN-β treatment is a relevant mechanism in linking KP activity with acute depressive symptoms in light of these findings (Amirkhani et al., 2005; Guillemin et al., 2001). Inflammation in suicidal depression is indicative of TRP metabolism shunting towards the KP with evidence of higher IDO activity in adolescents with major depression compared to controls and significant correlations between KYN/TRP ratio and level of suicidality in the unmedicated participants (Bradley et al., 2015). IDO activity and markers of inflammation are correlated in clinically depressed patients with a history of suicide attempts, bridging inflammatory and KP mechanisms together in suicidality (Sublette et al., 2011). Given the high risk of suicidality in MS and the evidence of shared inflammatory mechanisms, this information highlights the vulnerability of pwMS to more severe depressive symptoms as a result of the pathological inflammation in autoimmune diseases.

Additionally, activation of the KP and exploration of downstream metabolites demonstrates that similar KP abnormalities exist in both suicidality and MS progression. In line with IDO induction during inflammatory states such as MS relapse or PPD, higher KYN/TRP ratios are found in people with suicidal ideation and attempters compared to depressed controls (Sublette et al., 2011). Further, the degree of suicidal intent is significantly associated with QA, with its neurotoxic levels peaking at the time of attempt (Erhardt et al., 2012; Bay-Richter et al., 2015). Neither KYN nor QA are correlated with depression severity, suggesting an independent relationship between altered KP activity and suicide (Sublette et al., 2011; Erhardt et al., 2012). Suicide attempters have higher CSF QA/KA ratios compared to healthy controls, and this neurotoxic-indicative ratio was also found in MS progression (Lim et al., 2017; Bay-Richter et al., 2015). Post-mortem analysis of suicide victims (with depression and bipolar) have localised excitotoxic properties of NMDA agonist QA in the subgenual and anterior midcingulate of the anterior cingulate cortex (ACC), which contains a high density of NMDA receptors and is responsible for motivation (Steiner et al., 2011). This warrants similar post-mortem research in MS; if QA abundance can be localised in the CNS, this may open more treatment options such as ketamine, a NMDA receptor antagonist, which has been trialed successfully as an antidepressant for suicidality and treatment resistant depression (Serafini et al., 2014). This will enable future research to better understand QA within the context of suicidality in MS, as targeted treatment options for neuropsychiatric symptoms are needed to complement current disease modifying drugs.

### 5.2 Summary of suicide in MS

It is evident that the pathological components of inflammation and KP dysregulation (KP activation and QA accumulation) in depression can also be independently involved in suicide. Given the autoimmune nature of MS, this offers a plausible explanation of increased suicide risk in MS. This provides links to new opportunities in MS research to explore treatment options that mitigate QA-induced neurotoxicity through NMDA receptor antagonism.

## 6 Fatigue in MS

Fatigue affects at least 75% of pwMS (Lerdal et al., 2007), being the most common and debilitating symptom. Fatigue is prevalent even in the early stages of MS and ubiquitously affects physical and psychological domains such as energy, cognition, motivation and motor function. As such, it is unsurprising that fatigue is related to decrements in all aspects of quality of life and is central in limiting activities of daily living, particularly maintaining full time employment. Fatigue in MS is associated with a higher prevalence and severity of depression and this relationship is more consistent than links between fatigue and level of disability (Fassbender et al., 1998; Bakshi et al., 2000). The coexistence of these symptoms even when considering factors such as disability suggest that they may be secondary products of the mechanisms underlying MS disease progression (Bakshi et al., 2000).

### 6.1 Inflammation and fatigue in MS

The relationship between inflammation and fatigue provides evidence that it is a manifestation of cytokine-induced sickness behaviour in MS. Interestingly, while fatigue has been established as independent from disability, TNF-α is still moderately related to both fatigue and disease activity, providing evidence of a shared cytokine mediated basis (Heesen et al., 2006). PwMS experiencing fatigue have a higher capacity for TNF-α and IFN-γ stimulated production compared to non-fatigued MS controls (Heesen et al., 2006). Similarly, IL-6 has also been identified as a strong predictor as it accounts for a substantial portion (21%) of self-reported fatigue in pwMS without depression or sleep disorders (Malekzadeh et al., 2015). Further, immunomodulatory treatments that reduce pro-inflammatory cytokine levels correspondingly improve fatigue (Svenningsson et al., 2013). This provides a more direct role of proinflammatory cytokines in MS fatigue.

### 6.2 KP dysregulation and fatigue in MS

KP dysfunction can contribute to symptoms of fatigue in MS by disrupting mitochondrial function as the de novo synthesis of NAD+ ​through the KP is essential in several catabolic and enzymatic reactions to produce adenosine triphosphate (ATP). The reduction of NAD+ (to NADH) and oxidation of NADH (to NAD+) is required to maintain energy production processes in mitochondrion. Notably, pwMS have significantly reduced NAD+ and NAD+/NADH ratios in comparison to healthy controls, indicative of mitochondrial dysfunction (Lim et al., 2017; Braidy et al., 2013). NAD+ ​can be further depleted by reactive oxygen species (ROS), generated from major sources including pro-inflammatory cytokines (IFN-γ) and mitochondrial dysfunction. These reductions are in line with net QA elevations in MS (Lim et al., 2017) and additionally, EAE research has demonstrated that the catabolism of QA to NAD+ is limited, supporting the fact that pathological accumulation of QA is further preventing NAD ​+ ​synthesis in MS (Fig. 1) (Lim et al., 2017; Sundaram et al., 2020). This means that the mitochondrial functions that depend on NAD+, such as the generation of ATP for energy, are compromised in MS.

The impact of mitochondrial dysfunction affects clinical fatigue in MS through a neurodegenerative pathway. For example, lesion sites in the upper motor cortex require more energy for nerve conduction, but also have a reduced capacity to produce ATP (Dutta et al., 2006). To manage the discrepancy between ATP supply and demand, active lesion sites recruit more mitochondrion to support proper conduction (Witte et al., 2009). Indeed, increased electrical firing in MS lesions of the motor cortex during low-intensity movements are linked to quick fatigability, meaning that symptomatic fatigue in MS may be a result of an over exhaustion of energy expenditure (Ng et al., 1997). The pitfall of enhanced mitochondrial density is the production of oxidative stress and free radicals which perpetuate axonal degeneration, the putative cause of irreversible neurological decline in MS. This leads to further ATP depletion and an increase in ROS production, leading to a vicious cycle of collateral damage (Witte et al., 2009).

The ability to support mitochondrial functioning to balance levels of energy production with expenditure may help mitigate levels of fatigue in MS and in turn, slow the disease process. There is treatment potential in enhancing NAD+ ​precursors within the KP through vitamin B2 (riboflavin) and B3 (nicotinamide) supplementation to facilitate the de novo synthesis of ATP (Penberthy and Tsunoda, 2009). Daily infusions of vitamin B3 in mice after EAE induction delayed the development of symptoms and provided significant neurological and behavioural improvements compared to controls (Kaneko et al., 2006). Calorie restriction in mice can also regulate energy metabolism by raising NAD+ ​levels to protect against EAE (Piccio et al., 2008). As such, increasing NAD+ ​through various means is beneficial for clinical outcomes in animal models of MS. In humans, vitamin B2 and B3 supplementation on basal therapy and corticosteroids in MS participants reduced the need for corticosteroid therapy and improved EDSS scores (Bisaga et al., 2012). The efficacy of vitamin B complex supplementation in the progressive phases of disease are limited (Cree et al., 2020), which may highlight the utility of KP modulation as an early intervention option to protect against disease progression.

### 6.3 Summary of fatigue in MS

Fatigue is closely linked to depression in MS, and there is evidence of a cytokine mediated basis. The reduction of NAD+ ​due to KP dysregulation can disrupt mitochondrial function, reduce ATP output and increase ROS production. Together, these factors create energy imbalances that contribute to physical fatigue and encourages axonal degeneration. However, KP modulation through supplementation of Group B vitamins (B2 & B3) can improve NAD+ ​synthesis to overall stabilise mitochondrial function and ATP levels.

## 7 Cognitive dysfunction in MS

Across clinic and community based studies, cognitive dysfunction is prevalent in 40–65% of pwMS (Amato et al., 2006) and negatively affects employment status and engagement in social, recreational and household activities (Rao et al., 1991). Cognitive dysfunction in MS often presents as a heterogenous array of subtle declines across the domains of attention, processing speed, memory and executive functions, with more severe deficits in secondary and primary progressive MS. The most common cognitive deficits in pwMS fall under information processing speed deficiencies (Denney et al., 2004) that may limit higher order cognitive functions such as working memory (the ability to hold and manipulate information) and episodic memory (remembering new information). For example, pwMS may take longer to complete more complex or demanding tasks, but perform equivalently to healthy controls on similar untimed measures (Denney et al., 2004).

Interestingly, the presence of depression and fatigue can exacerbate cognitive deficits in MS (Denney et al., 2004). Depression mediates the relationship between performance on tasks of processing speed, verbal fluency, working memory and memory recall (Landrø et al., 2004; Diamond et al., 2008). Indeed, pwMS who have elevated symptoms of depression perform significantly worse than matched non-depressed pwMS and healthy controls on tasks of working memory, planning efficiency, information processing, cognitive flexibility and psychomotor speed (Arnett et al., 1999, 2001). This supports the idea that mood has a significant secondary effect on cognitive tasks that tap multiple operations.

Due to the direct links between neurodegeneration and progressive cognitive decline, it is relevant to explore the neurodegenerative pathway that stems from inflammatory and KP mechanisms in cognition in MS that may impact structural and functional changes in the brain and contribute to the cognitive deficits discussed above.

### 7.1 Inflammation and cognitive dysfunction in MS

There are strong links between inflammation and cognitive deficits in MS. Indeed, memory decline is associated with early immune activation and inflammation (IL-1β and TNF-α) in EAE mice before the onset of other neurological symptoms or demyelination (Acharjee et al., 2013). Studies on pwRRMS have demonstrated that cytokine levels of IL-6, TNF-α and IL-17 are associated with poorer performance on a global cognitive screener (Patanella et al., 2009). A reduction in working memory performance over time in pwRRMS also seems to be contingent on the presence of active lesions on MRI, suggesting the role of focal inflammation on cognitive function (Bellmann-Strobl et al., 2009). Remarkably, cognitive remediation has been achieved through disease modifying drugs with anti-inflammatory properties; administration of glatiramer acetate in EAE mice was able to conserve some memory impairments (Aharoni et al., 2019). Human trials of anti-inflammatory disease modifying drugs in RRMS also seem to reduce cognitive decline (Iaffaldano et al., 2016). These successful intervention studies provide more direct evidence of an inflammatory role in mediating cognitive outcomes in MS.

There is also evidence that inflammation drives demyelination. The pathological hallmark of MS is multiple focal areas of demyelination within the CNS, and this susceptibility of white matter regions is the primary mechanism underlying cognitive deficits. Pro-inflammatory cytokines, especially TNF-α, can alter cell signalling to induce oligodendrocyte cell death and demyelination in mice (Akassoglou et al., 1998). Further, remyelination is also more difficult during pro-inflammatory states as proliferation and differentiation of oligodendrocyte progenitor cells into oligodendrocytes to rebuild myelin is compromised (Moore et al., 2015). As such, the glial cells supporting myelin are destroyed, catalysing lesion formation in white matter while remyelination is prevented to sustain white matter damage in MS. White matter changes are more extensive in individuals with MS who have cognitive impairment compared to those who are cognitively intact (Hulst et al., 2013). Reduced functional activation has also been demonstrated in frontal white matter tracts, which are associated with poorer performance on measures of working memory (Au Duong et al., 2005). More specifically, weaker functional connectivity between the ACC and prefrontal regions during working memory tasks has been identified even in the prodromal phases of MS (Au Duong et al., 2005). Interestingly, the ACC overlaps as a region implicated in both cognitive dysfunction in MS and QA excitotoxicity in suicide. If the ACC is implicated so early in the disease course, this highlights the susceptibility of this area to inflammatory conditions such as depression and MS. Another targeted region appears to be the hippocampus, as pro-inflammatory cytokines (TNF-α and IL-1β) can also alter excitatory and inhibitory synaptic transmissions in the hippocampus, causing memory and learning deficits in EAE (Habbas et al., 2015; Nisticò et al., 2013). Conversely, a reduction of pro-inflammatory cytokines and downregulation of T cell infiltration in EAE mice following immunomodulation was shown to protect against spatial learning deficits (Aharoni et al., 2019). These studies implicate inflammation in the early vulnerability of certain brain regions such as the ACC and hippocampus in underlying cognitive dysfunction in areas of processing speed, working memory, learning and memory due to the brain’s compromised ability to communicate between and within regions in MS.

### 7.2 KP dysregulation and cognitive dysfunction in MS

As described in section 2.2, several downstream KP metabolites are neuroactive and have been linked to neurodegeneration, especially QA. Several studies have reported increased QA in neurodegenerative diseases including MS, Alzheimer’s disease, HIV and Huntington’s disease (Lovelace et al., 2017). For example, the neurotoxin QA is found in higher concentrations in the plaques and tangles of post-mortem hippocampal tissue in Alzheimer’s disease (Guillemin et al., 2005). Further, QA in the CNS is abundant in people with HIV and is linked to neuropsychological deficits including processing speed, memory and learning (motor, verbal and non-verbal), language and verbal fluency (Heyes et al., 1991). In Huntington’s disease, QA administration in animal models produces working memory, spatial learning and motor coordination deficits which are also apparent during EAE induction (Block et al., 1993). These converging findings implicate KP as the strongest link in explaining why neuroinflammation may lead to neurodegeneration and highlight a related pattern of associated cognitive deficits. As such, there is an unmet need to better understand the relationship between QA levels and cognitive functioning in MS.

Brain regions related to neuropsychological dysfunction in MS are highly sensitive to pathophysiological levels of QA. Selective neurotoxicity within the hippocampus and striatum has been demonstrated following QA induction in rats (Schwarcz and Köhler, 1983). Similarly, the hippocampus demonstrates reduced synaptic density, hippocampal apoptosis and volume loss in EAE that is associated with spatial memory and learning deficits compared to control mice (Ziehn et al., 2010). While localisation of QA concentrations have yet to be examined in post-mortem MS studies, there is evidence of a disproportionate loss of hippocampal volume compared to global brain atrophy in MS that is indicative of the selectivity of QA neurodegeneration in this area (Sicotte et al., 2008). The pattern of extensive hippocampal atrophy has also been identified in people with RRMS and secondary progressive MS and are related to reduced encoding on tasks of memory (Sicotte et al., 2008). Not only is hippocampal volume (particularly the dentate gyrus) predictive of MS diagnosis during the clinically isolated syndrome phase, but the size of this region is related to ongoing atrophy and memory recall performance one year later (Planche et al., 2018), suggesting that KP mechanisms are operating early on in the disease.

QA abundance has also previously been established in the ACC in people who died of completed suicide (Steiner et al., 2011) and consistent with this, the focal lesion load in the ACC of pwRRMS is higher than what is expected from normal aging (Charil et al., 2007). Notably, the ACC is also related to performance on executive tasks (timed verbal strategy generation and inhibition), with pwMS performing markedly worse in both these tasks compared to healthy controls (Denney et al., 2004). This suggests that QA selectively damages important brain regions involved in memory, executive function and reward, where NMDA receptors are saturated (Guillemin et al., 2005). The patterns of neurodegeneration in MS are consistent with QA-induced neurotoxicity, however, the direct relationship with QA and cognitive function has yet to be established in MS research.

### 7.3 Summary of cognition in MS

The cognitive profile in MS is characterised by impairments in processing speed, working memory, executive functions and learning and memory. These deficits can be explained by inflammatory mechanisms contributing to white matter damage and synaptic alterations in the prefrontal cortex, ACC and hippocampal regions in the brain. A pattern between neuroinflammation and abnormal QA accumulation has also been identified leading to increased levels of excitotoxicity in brain regions such as the hippocampus and the ACC where there is a saturation of NMDA receptors. However, it is evident that research in this area is limited and the extent and severity of QA excitotoxicity on cognition in MS requires further clarification.

## 8 Concluding summary

**Fig. 2:**
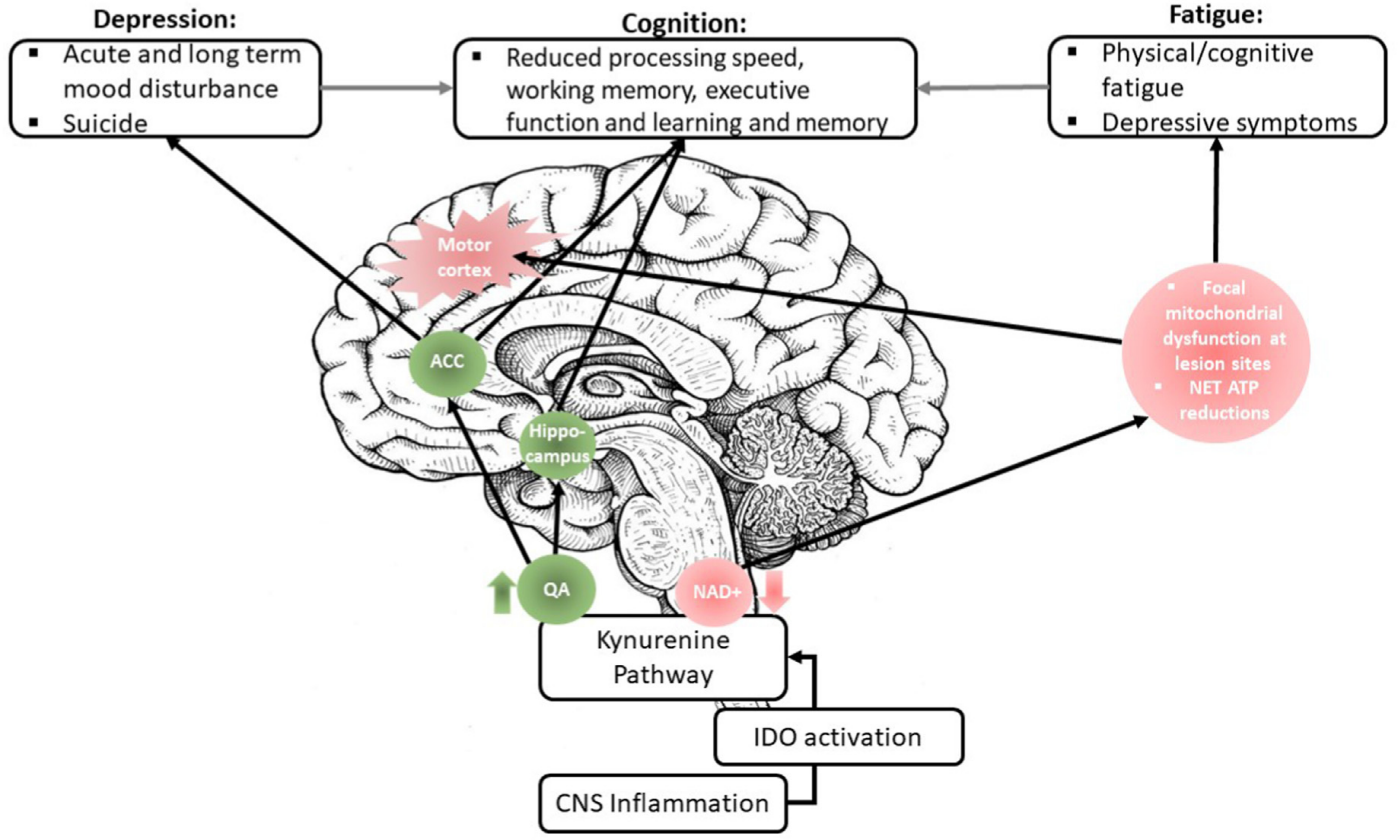
The neuropsychiatric consequences of KP metabolites in the CNS. This diagram provides a more focal depiction of KP dysregulation in the brain. The increase (green) of QA has been localised in the ACC (in KP suicide literature) and the hippocampus (in postmorterm studies of Alzheimer’s disease). Interestingly, atrophy within these regions are disproportionately higher in MS compared to other regions, which may imply KP mechanisms in these areas, causing depression and cognitive dysfunction in MS. Correspondingly, QA abundance in MS precludes the biosynthesis of NAD+, overall leading to net ATP reductions (red), particularly at active lesion sites in the motor cortex that require large amounts of energy. The unmet need of energy expenditure underlies levels of fatiguability in MS, which are closely related to depressive symptoms. The grey arrows also demonstrate how the symptoms of both depression and fatigue can secondarily impact cognition by compounding on processing speed deficits. (For interpretation of the references to colour in this figure legend, the reader is referred to the Web version of this article.)

The aim of this review was to provide a better understanding of common and overlooked neuropsychiatric symptoms in MS (depression, PPD, suicide, fatigue and cognitive dysfunction) from a perspective that encompasses the evolving roles of inflammation and TRP metabolism. It is evident that the duality between pro-inflammatory cytokines and KP dysregulation can perpetuate disease progression and exert secondary effects on mood, energy balances and cognition. Pro-inflammatory states sustain these symptoms through cytokine-induced sickness behaviours and catalyse neurodegenerative processes by activating the neurotoxic arm of the KP. This review integrated how these processes affect neurotransmitter depletion (5HT and melatonin), mitochondrial function and structural and functional changes in the brain to produce neuropsychiatric symptoms in MS (Fig. 1). There was also a focus on converging evidence to argue for the direct role of KP metabolites, specifically the effects of increases in QA and decreases in NAD+ in MS (Fig. 2). This provides strong evidence of the dysfunctional nature between inflammation and KP metabolism in MS, offering a plausible mechanism that explains the prevalence neuropsychiatric symptoms that occur in the background of disease progression.

The consequences of inflammation and altered KP metabolism on neuropsychiatric symptoms allows us to recognise the broader impact of these mechanisms, which affect more than just disease progression variables, but include everyday functioning and quality of life for pwMS. In turn, research on these mechanisms can provide insight into a more holistic yet personalised approach for treatment management options in MS. Potential treatments that modulate KP function through various means – diet, supplementation, novel anti-depressants and other pharmacotherapies were explored here, however these efforts require a more specific focus in MS research. Other than minimising levels of inflammation, appropriate levels of IDO activation require elucidation regarding the balance between immunosuppression and KP-induced neurodegeneration. This review highlights the unmet need for future treatment options that target KP regulation. The ‘optimal’ balance at which relapse can be suppressed by IDO induction while averting downstream KP dysfunction, thereby slowing or halting MS progression has yet to be unveiled. Hence, there are prospects to advance the development of KP biomarkers in relation to disease burden to monitor disease progression, which would complement the development of treatments to prevent or reverse the neurodegenerative stages of disease. Such biomarker-guided treatments are urgently needed and can also directly benefit cognitive, behavioural and functional outcomes in MS.

## Author contributions

C.K.L. developed the original idea, conceptual framework and structure for the review. L.T. conducted the literature review, designed the figures and wrote the manuscript. C.K.L. and H.F. were both involved in editing of the figures and manuscript. All authors reviewed the final manuscript.

## Declaration of competing interest

The authors declare that they have no conflict of interests.
